# Perioperative virtual reality for pain and anxiety in adult hand and wrist surgery: a systematic review

**DOI:** 10.1016/j.jpra.2026.09.005

**Published:** 2026-09-12

**Authors:** Shivam Bhindi, Thanmay Mangala Sachidananda, Ali Alseneid, Gunjeet Dua

**Affiliations:** a Faculty of Life Sciences and Medicine, King’s College London, Guy’s Campus, London SE1 1UL, United Kingdom; b Department of Internal Medicine, William Harvey Hospital, Kennington Road, Willesborough, Ashford, Kent TN24 0LZ, United Kingdom; c Department of Anaesthesia, Guy’s and St Thomas’ NHS Foundation Trust, Great Maze Pond, London SE1 9RT, United Kingdom

**Keywords:** Virtual reality, Hand and wrist surgery, Wide-awake local anaesthesia no tourniquet, Perioperative anxiety, Perioperative pain, Systematic review

## Abstract

**Introduction:**

Many adult hand and wrist procedures are performed with the patient awake under local or regional anaesthesia, including wide-awake techniques, a setting in which perioperative anxiety is a salient patient-experience outcome and discomfort may persist despite adequate anaesthesia. Virtual reality has been proposed as a non-pharmacological comfort adjunct, but its effects are procedure-dependent and existing trials come largely from other settings; the high volume of awake hand and wrist surgery, where patients are conscious during anaesthetic administration and manipulation, makes procedure-specific evidence necessary, and none has been synthesised.

**Methods:**

MEDLINE (Ovid), Embase and CENTRAL were searched from inception to 19 December 2025 for randomised controlled trials of perioperative virtual reality in adult hand and wrist surgery (PROSPERO CRD420251269092). Selection and extraction were performed in duplicate. Risk of bias was assessed with Cochrane Risk of Bias 2 and certainty with GRADE. Heterogeneity precluded meta-analysis; findings were synthesised narratively.

**Results:**

Seven trials (n = 543; 2019–2025) met inclusion criteria. Virtual reality did not consistently reduce perioperative pain. Of six trials assessing anxiety, three reported significant reductions in intraoperative anxiety during awake procedures; instruments and time points were heterogeneous, and the wide-awake local anaesthesia no tourniquet trial using a validated multi-item questionnaire showed no significant difference. Two trials reported lower intraoperative sedative requirements in pathways including sedation. Virtual reality was well tolerated, with no serious adverse events and minor self-limiting effects in a minority of participants. Certainty of evidence was low for both primary outcomes.

**Conclusion:**

Virtual reality may reduce intraoperative anxiety in selected patients undergoing awake hand and wrist procedures, but evidence for analgesic benefit is inconsistent. Larger trials using validated anxiety instruments, stratified by baseline anxiety, are needed before routine use can be recommended.

## Introduction

Pain and anxiety are common perioperative concerns for adults undergoing hand and wrist surgery, particularly during awake procedures performed under local or regional anaesthesia.^1^^,^^2^ Contemporary hand and wrist surgery practice increasingly emphasises outpatient and procedure-room pathways, including wide-awake techniques, driven by efficiencies in care delivery and patient experience.^3^, ^4^, ^5^ In these awake pathways, intraoperative anxiety becomes a meaningful patient-experience outcome in its own right, and clinically relevant preoperative anxiety has been reported in adults undergoing ambulatory hand surgery.^1^

Virtual reality (VR) is a non-pharmacological intervention that uses immersive audiovisual environments to reduce pain and anxiety through distraction and attentional engagement.^6^^,^^7^ Across perioperative and procedural settings, VR has been evaluated as an adjunct to reduce anxiety, procedural discomfort and analgesic or sedative requirements; however, reported effects vary substantially by procedure and implementation, underscoring the need for procedure-specific evaluation.^6^^,^^8^

Hand and wrist surgery represents a pragmatic setting for VR because many procedures are performed with patients awake, creating opportunities to address anxiety during anaesthetic administration, intraoperative manipulation and early recovery. An early observational study of VR in wide-awake hand surgery reported feasibility and patient acceptability,^9^ but randomised trials have demonstrated variable effects on pain and anxiety across outcomes and perioperative time points.^10^, ^11^, ^12^

Despite growing interest in perioperative VR, evidence remains limited by small sample sizes, heterogeneous methodologies and inconsistent outcome measures.^6^ To our knowledge, no systematic review has previously synthesised the randomised evidence specifically in adult hand and wrist surgery. This review evaluates the effects of perioperative VR on pain and anxiety in this population compared with standard care; secondary objectives are effects on patient satisfaction, analgesic or opioid consumption, physiological measures and VR-related adverse events.

## Methods

### Protocol and registration

This review was conducted and reported in accordance with the Preferred Reporting Items for Systematic Reviews and Meta-Analyses (PRISMA) 2020 guidelines. The protocol, including prespecified eligibility criteria and outcomes, was prospectively registered with PROSPERO (CRD420251269092). Although meta-analysis was prespecified, quantitative synthesis was not performed owing to substantial clinical and methodological heterogeneity in interventions, outcome measures and assessment timing across included studies. A completed PRISMA 2020 checklist is provided as Supplementary Material.

### Search strategy

A literature search was conducted in MEDLINE (via Ovid), Embase and the Cochrane Central Register of Controlled Trials (CENTRAL) from inception to 19 December 2025. An update search on 16 May 2026 using the same strategy identified no additional eligible studies. The strategy combined database-specific controlled vocabulary (e.g. MeSH and Emtree terms) with free-text keywords related to virtual reality and hand or wrist surgery using Boolean operators; it was designed for high sensitivity within surgical, anaesthetic and randomised trial databases and was not restricted by outcome terms. No language restrictions were applied; all eligible studies identified were published in English. In addition, reference lists of included studies were manually screened, and Google Scholar was screened through 19 December 2025 (first 200 results) to identify eligible randomised controlled trials (RCTs) not indexed in the selected databases. Full search strategies are provided in the Supplement.

### Eligibility criteria

Eligible studies were RCTs enrolling adults (≥18 years) undergoing hand or wrist surgery, including elective and trauma-related procedures, as registered in our PROSPERO protocol. Eligibility was not restricted by pathology, tissue type or operative technique: soft-tissue, bony and arthroscopic procedures, including distal radius fixation and wrist arthroscopy, were all eligible, the only surgical exclusion being procedures not involving the hand or wrist. The adult-only restriction reflects the distinct anaesthetic, consent and perioperative considerations in paediatric hand surgery and ensures clinical homogeneity. Perioperative VR interventions delivered before, during or immediately after surgery using immersive or semi-immersive head-mounted displays were included. Comparators consisted of standard perioperative care with or without non-VR distraction. Any anaesthetic modality was eligible, provided participants remained conscious during VR exposure. Studies evaluating VR solely for surgical training, simulation or postoperative rehabilitation were excluded.

### Outcomes

Primary outcomes were prespecified patient-reported pain and anxiety, measured using validated or study-specific instruments. Secondary outcomes were analgesic and sedative consumption, physiological measures, patient satisfaction and VR-related adverse events, assessed at preoperative, intraoperative or postoperative time points where reported. For primary outcomes, patient-reported pain and anxiety were prioritised over observational or physiological proxies. When multiple measures or time points were reported, we extracted the prespecified primary endpoint; if none was prespecified, we extracted the outcome measured during active VR exposure that most directly reflected patient-reported perioperative pain or anxiety. Observational pain tools, graphical reporting and study-specific scales were interpreted as lower-confidence evidence. Clinically actionable secondary outcomes (e.g. sedative or opioid requirements, recovery metrics, physiological measures) were selectively tabulated, while all reported outcomes were considered in the narrative synthesis.

### Study selection and data extraction

All records were imported into Rayyan for deduplication and screening. Two reviewers independently screened titles and abstracts, followed by full-text review of potentially relevant studies; disagreements were resolved by discussion and consensus, with a third reviewer available but not required. Data were extracted using a prespecified structured form developed for this review (available from the corresponding author on request), covering study design, sample size, participant demographics, surgical and anaesthetic context, characteristics and timing of the VR intervention, comparator details, and all reported outcomes relevant to pain, anxiety and secondary endpoints. Extraction disagreements were resolved by discussion and consensus. When outcome data were reported only graphically or were not extractable, corresponding authors were contacted to request numerical values; no additional data were obtained. Where participants were excluded from specific outcome analyses, the number analysed for that outcome was extracted rather than the total randomised sample.

### Risk of bias assessment

Risk of bias for each included RCT was assessed independently by two reviewers using the Cochrane Risk of Bias 2 tool across five domains: randomisation process, deviations from intended interventions, missing outcome data, measurement of the outcome, and selection of the reported result. Each study was rated as low risk, some concerns or high risk, at the outcome level and separately for pain and anxiety where applicable. Detection bias was captured within the measurement-of-outcome domain, with attention to whether outcome assessors were blinded to allocation and whether outcomes were patient-reported or observer-rated.

### Certainty of evidence assessment

Certainty of evidence for pain and anxiety outcomes was assessed using the GRADE Working Group approach, with judgements informed by risk of bias, inconsistency, indirectness, imprecision and publication bias. Owing to heterogeneity in outcome measures and assessment time points, certainty judgements were documented using GRADEpro GDT and summarised narratively.

### Data synthesis

Although quantitative synthesis was prespecified, meta-analysis was not feasible because of substantial clinical and methodological heterogeneity across studies, including variation in VR interventions, surgical settings, outcome measures and timing of assessment, and incomplete reporting of summary statistics required for pooled effect estimates. Publication bias was not formally assessed because the small number of studies and absence of quantitative synthesis precluded meaningful evaluation. Findings were synthesised using a structured narrative approach, grouping studies by outcome (pain and anxiety), perioperative time point and direction of effect, and highlighting patterns of consistency and sources of heterogeneity. Where different outcome instruments were used, results were interpreted within the context of each instrument and were not pooled across scales.

## Results

### Study selection

The search identified 474 records; after duplicate removal, 335 remained. Eleven reports underwent full-text assessment, of which six RCTs met eligibility through database searching and five were excluded (reasons in the PRISMA 2020 flow diagram, Fig. 1). One further eligible trial was identified by manual searching, giving seven included trials.^10^, ^11^, ^12^, ^13^, ^14^, ^15^, ^16^

**Fig. 1:**
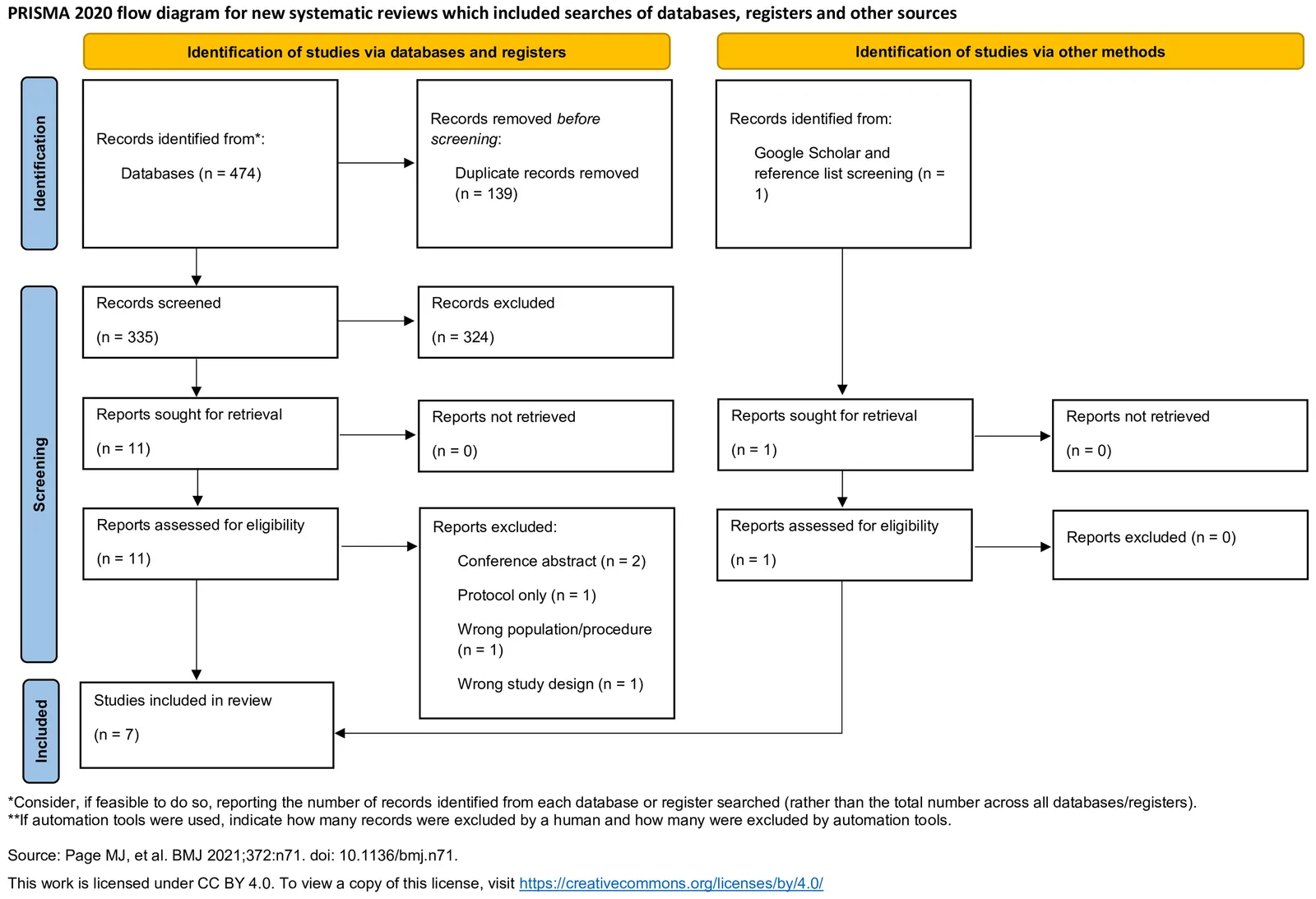
PRISMA 2020 flow diagram of study selection. Records were identified through database searching of MEDLINE (Ovid), Embase and CENTRAL and through manual searching. After duplicate removal, titles and abstracts were screened for eligibility, followed by full-text review. Reasons for exclusion at full-text stage are indicated. Seven randomised controlled trials met the inclusion criteria and were included in the qualitative synthesis.

### Study characteristics

The seven trials comprised 543 adults undergoing hand and wrist procedures across outpatient, procedure-room and emergency department settings (Table 1). VR was delivered using immersive or semi-immersive head-mounted displays at varying time points, from consumer headsets running commercial software to a medical-grade hypnotic system in one trial. Pain and anxiety were assessed with heterogeneous patient-reported instruments, with observational pain measures and physiological variables in a subset of trials (Tables 2 and 3; Supplements S1 and S2). No clear selective outcome reporting was identified, though formal assessment of publication bias was limited by the small number of studies.

**Table 1:** Characteristics of included randomised controlled trials.

Study	Sample size (VR/control)	Age, y (VR/control)	Surgical setting / procedure	Anaesthesia context	VR intervention (device / content; timing)	Comparator	Primary outcomes measured
Hoxhallari et al., 2019	21 / 20	20–82 / 38–82 (range)	WALANT hand surgery	Local anaesthesia with adrenaline	Samsung Galaxy S7 with Samsung Gear VR; pre-recorded immersive 3D/360 content; intraoperative	Standard WALANT care without VR	Pain (10-point Likert, 0–10); anxiety (10-point Likert, 0–10)
Faruki et al., 2022 [a]	17 / 17	48.7 (SD 19.0) / 50.1 (SD 11.1) (mean, SD)	Elective hand surgery	Regional anaesthesia with monitored anaesthesia care	Oculus Go with noise-cancelling headphones; patient-selected audiovisual content; intraoperative	Usual MAC / standard perioperative care without VR	Primary endpoint of original trial was intraoperative propofol per hour; pain and anxiety measured as secondary endpoints
Gharanli et al., 2024	80 / 80	33.1 (SD 11.9) / 31.0 (SD 10.3) (mean, SD)	Emergency department hand laceration repair	Local anaesthesia with lidocaine	Semi-immersive VR headset with mobile phone; natural-scene audiovisual content; intraoperative	Standard local anaesthetic care without VR	Pain (VAS 0–10; CPOT); anxiety not assessed
Nijs et al., 2025 [b]	59 / 59	55.5 (45.3–59.0) / 52.5 (45.3–59.0) (median, IQR)	Elective hand surgery	Ultrasound-guided regional anaesthesia	Sedakit VR glasses (Oncomfort) medical-grade hypnotic VR; preoperative and intraoperative	Standard regional anaesthesia care without VR	Pain (NRS 0–10); anxiety (NRS 0–10)
McConnell et al., 2025	45 / 45	63 (48–67) / 63 (57–68) (median, IQR)	WALANT hand surgery	Wide-awake local anaesthesia (WALANT)	Meta Quest 2; YouTube VR app, "Our Planet" videos; intraoperative	Standard WALANT care without VR	Pain (PROMIS Pain Interference, preoperative to 2-week postoperative change); anxiety (APAIS)
Xiao et al., 2025	20 / 21	46.3 (24–75) / 57.5 (23–73) (mean, range)	Minor outpatient hand surgery	Local anaesthesia with or without sedation (monitored anaesthesia care)	Oculus Go; AppliedVR software; immersive landscape content; preoperative and intraoperative	Standard perioperative care without VR	Pain (NRS 0–10); anxiety (NVAAS 0–10)
Matsuoka et al., 2025	30 / 29	64 (SD 15) / 62 (SD 14) (mean, SD)	Minor elective hand surgery	Wide-awake local anaesthesia (WALANT)	WAVR (Wide Awake Virtual Reality) platform; preoperative through completion of surgery	Standard WALANT care without VR	Pain (Wong-Baker FACES 0–10); anxiety (VAS 0–10)

[a] Forty participants were randomised; 34 completed perioperative outcome assessment.

[b] Sixty per arm were randomised; 59 per arm were analysed in outcome tables.

VR, virtual reality; WALANT, wide-awake local anaesthesia no tourniquet; MAC, monitored anaesthesia care; VAS, visual analogue scale; NRS, numerical rating scale; CPOT, Critical-Care Pain Observation Tool; APAIS, Amsterdam Preoperative Anxiety and Information Scale; PROMIS, Patient-Reported Outcomes Measurement Information System; NVAAS, Numerical Visual Analog Anxiety Scale; IQR, interquartile range; SD, standard deviation.

### Risk of bias within studies

Risk of bias varied across trials (Fig. 2). One trial was at low risk across all domains.^16^ Four had some concerns, most often from deviations from the intended intervention, the randomisation process and outcome measurement.^11^, ^12^, ^13^^,^^15^ Two were at high risk — Gharanli et al.^14^ for pain and Hoxhallari et al.^10^ for both pain and anxiety — primarily from missing outcome data; in Hoxhallari et al.,^10^ deviations from the intended intervention (including absence of blinding) also contributed. Blinding of participants was not feasible in any trial, and only Nijs et al.^16^ used an assessor blinded to allocation for pain and anxiety, so susceptibility to detection and reporting bias was a consistent limitation.

**Fig. 2:**
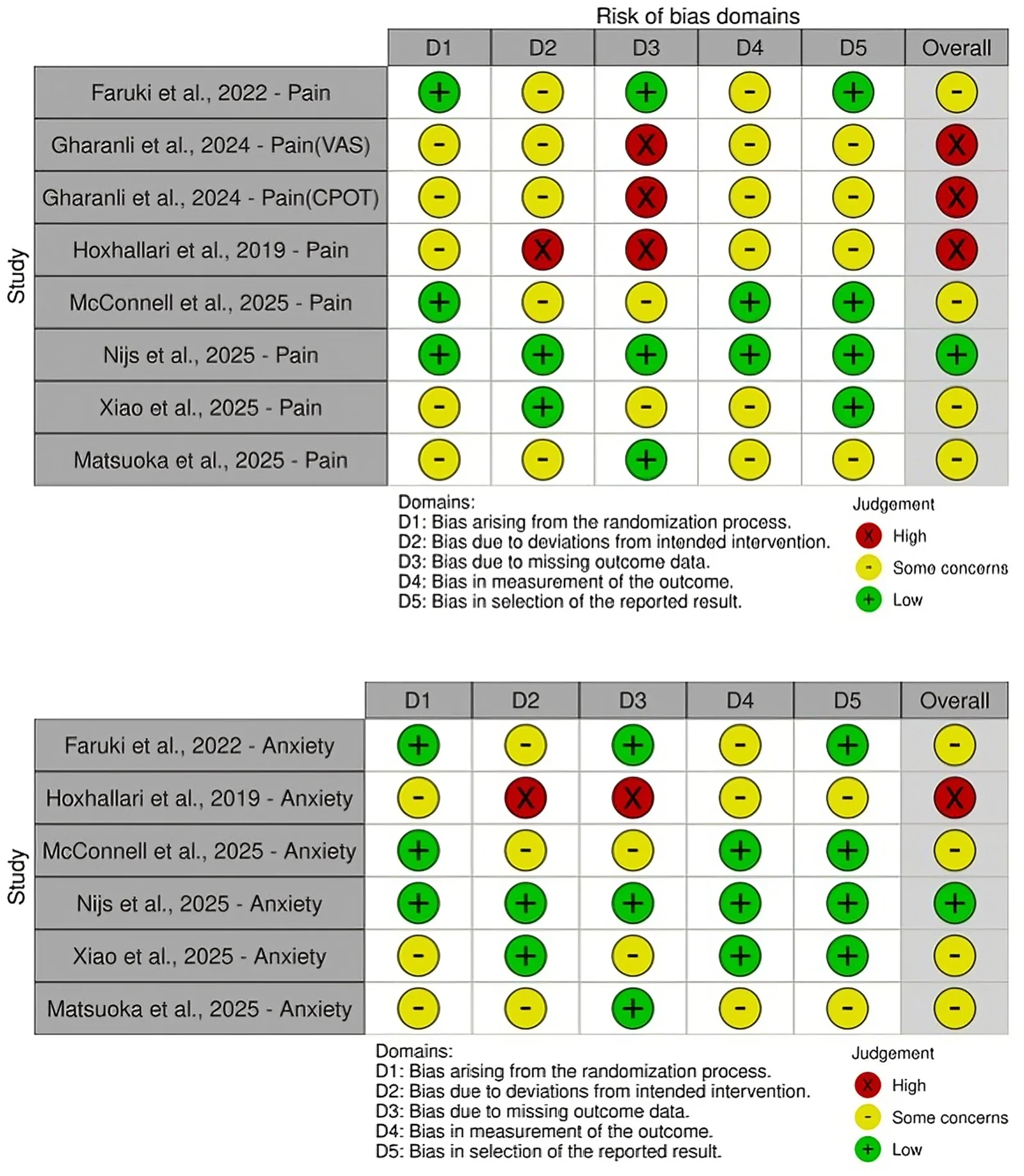
Risk of bias assessment of included randomised controlled trials using the Cochrane Risk of Bias 2 tool. Traffic-light plots summarise risk-of-bias judgements across domains for pain outcomes (top panel) and anxiety outcomes (bottom panel). Domains assessed included the randomisation process, deviations from intended interventions, missing outcome data, measurement of the outcome, and selection of the reported result. Green indicates low risk of bias; yellow indicates some concerns; red indicates high risk of bias. RCT, randomised controlled trial.

### Primary outcomes

Pain was measured at variable time points using different instruments (Table 2). During ultrasound-guided regional anaesthesia, pain at block placement did not differ between groups.^16^ Across elective outpatient surgery, perioperative pain did not differ significantly, whether under monitored anaesthesia care (MAC)^12^^,^^13^ or wide-awake local anaesthesia no tourniquet (WALANT).^11^^,^^15^ One WALANT trial found no overall difference but reported lower pain during local anaesthetic injection in a subgroup with self-reported pre-existing anxiety disorder; these data were graphical and not extractable, and the signal is exploratory.^10^ In the emergency department — differing from elective surgery in pathway, indication and intent — post-procedure pain was lower with VR.^14^

**Table 2:** Primary pain outcomes in included randomised controlled trials.

Study	N analysed (VR/control)	Pain instrument	Primary time point reported [a]	VR group result [b]	Control group result	Statistical comparison	Additional notes
Faruki et al., 2022	17 / 17	NRS (0–10)	PACU	0 (0–0)	0 (0–0)	p = 0.33	Intraoperative pain was reported separately using a study-specific 0–100 agreement-style scale
Gharanli et al., 2024	80 / 80	VAS (0–10)	Immediately after laceration repair	1.68 (SD 1.35)	2.45 (SD 0.88)	p < 0.001	Observational CPOT was also collected; conducted in an ED laceration repair population rather than elective hand surgery
Hoxhallari et al., 2019	21 / 20	10-point Likert pain scale (0–10)	Local anaesthetic injection	NR (reported graphically)	NR (reported graphically)	No extractable overall values	Post hoc subgroup with pre-existing anxiety: VR lower at injection (p = 0.02)
Matsuoka et al., 2025	30 / 29	Wong-Baker FACES (0–10)	Intraoperative	0.3	0.7	p = 0.25	Original paper reported raw 0–10 values and percentages of scale maximum; raw values are presented
McConnell et al., 2025	45 / 45	PROMIS Pain Interference	Change from preoperative baseline to 2-week postoperative follow-up	0.0 (–6.0, 5.0)	1.0 (0.0, 5.0)	p = 0.174	Captures pain-related functional interference rather than acute perioperative pain intensity
Nijs et al., 2025	59 / 59	NRS (0–10)	During ultrasound-guided regional block placement	3.6 (SD 2.4)	3.9 (SD 2.4)	Mean difference –0.3 (95% CI –1.2 to 0.5); p = 0.22	Prespecified primary pain endpoint in the original trial
Xiao et al., 2025	20 / 21	NRS (0–10)	Intraoperative	1.3 (0–5)	1.1 (0–7)	p = 0.74	Preoperative and PACU pain outcomes were also reported and were similarly non-significant

[a] Where a primary pain endpoint was not explicitly prespecified, intraoperative pain during active VR exposure was extracted. Where multiple pain measures were reported at the same time point, patient-reported pain intensity measures were prioritised over observational measures.

[b] Values are reported as mean (SD), median (IQR), or mean (range), as reported in individual studies.

CI, confidence interval; CPOT, Critical-Care Pain Observation Tool; ED, emergency department; IQR, interquartile range; NR, not reported; NRS, numerical rating scale; PACU, post-anaesthesia care unit; PROMIS, Patient-Reported Outcomes Measurement Information System; SD, standard deviation; VAS, visual analogue scale; VR, virtual reality.

Anxiety was assessed with heterogeneous instruments at different time points (Table 3). Six trials assessed anxiety; three reported significant intraoperative reductions with VR. Under regional anaesthesia, anxiety during surgery was lower with VR while anxiety at block placement did not differ.^16^ In one WALANT trial, intraoperative and postoperative anxiety were significantly lower with VR while preoperative anxiety did not differ.^15^ A further WALANT trial reported significantly lower anxiety at all three intraoperative time points (injection, mid-procedure, end of procedure) using a study-specific Likert scale, with values graphical and not extractable; a subgroup with self-reported pre-existing anxiety disorder showed additional benefit mid-procedure.^10^ By contrast, anxiety did not differ in a WALANT trial using the multi-item Amsterdam Preoperative Anxiety and Information Scale (APAIS).^11^ or in two outpatient trials under regional or local anaesthesia with MAC sedation.^12^^,^^13^ — though Faruki used a single-item scale, and Xiao reported numerically higher intraoperative anxiety with VR that was not significant.

**Table 3:** Primary anxiety outcomes in included randomised controlled trials.

Study	N analysed (VR/control)	Anxiety instrument	Primary time point reported [a]	VR group result [b]	Control group result	Statistical comparison	Additional notes
Faruki et al., 2022	17 / 17	0–100 agreement-style scale: "I felt anxious"	Postoperative recall of intraoperative experience	0 (0–30)	0 (0–2)	p = 0.36	Reported as median (IQR); single-item study-specific scale; the original paper contains an internal inconsistency between text and Table 3 p-values
Hoxhallari et al., 2019	21 / 20	10-point Likert anxiety scale (0–10)	Local anaesthetic injection	NR (reported graphically)	NR (reported graphically)	NR (graphical only)	Group differences reported as significant by the original authors at injection, mid-procedure and end of procedure (p ≤ 0.04) but presented graphically without extractable numerical means
Matsuoka et al., 2025	30 / 29	VAS (0–10)	Intraoperative	1.0 (95% CI 0.41–1.6)	4.1 (95% CI 2.9–5.4)	p = 0.01	Reported with 95% CIs rather than mean (SD); intraoperative time point selected to reflect anxiety during active VR exposure
McConnell et al., 2025	45 / 45	APAIS (maximum 30)	Change from preoperative baseline to immediate postoperative assessment	NR (median change)	NR (median change)	p = 0.315	No statistically significant between-group difference on the APAIS, a multi-item anxiety instrument
Nijs et al., 2025	59 / 59	NRS (0–10)	Intraoperative	0.0 (0.0–2.0)	1.5 (0.0–4.0)	p < 0.01	Median (Q1, Q3); anxiety during block placement was not significantly different
Xiao et al., 2025	20 / 21	NVAAS (0–10)	Intraoperative	3.1 (0–10)	1.7 (0–6)	p = 0.09	Mean (range); VR group numerically higher; preoperative and PACU anxiety also non-significant

[a] Where a primary anxiety endpoint was not prespecified, intraoperative anxiety during active VR exposure was extracted.

[b] Values are reported as mean (SD), median (IQR), mean (range), or mean with 95% confidence intervals, as reported in individual studies.

APAIS, Amsterdam Preoperative Anxiety and Information Scale; CI, confidence interval; IQR, interquartile range; NR, not reported; NRS, numerical rating scale; NVAAS, Numerical Visual Analog Anxiety Scale; PACU, post-anaesthesia care unit; VAS, visual analogue scale; VR, virtual reality.

### Secondary outcomes

Secondary outcomes were inconsistently reported. Sedative requirements were lower with VR in two trials: lower intraoperative propofol and shorter post-anaesthesia care unit stay under monitored anaesthesia care regional anaesthesia,^13^ and lower intraoperative fentanyl and midazolam under local anaesthesia with MAC sedation,^12^ perioperative opioid use did not differ in the regional anaesthesia trial.^16^ Physiological measures were reported in five trials with mixed findings: lower systolic blood pressure and respiratory rate in Gharanli et al.,^14^ lower intra- and postoperative heart rate in Matsuoka et al.,^15^ and a divergent intraoperative diastolic blood pressure change (a small rise in control versus a small fall with VR) in McConnell et al.,^11^ and no significant differences in Hoxhallari et al.^10^ or Xiao et al.^12^ Heart rate variability, an objective stress index in the regional anaesthesia trial, did not differ.^16^ Patient satisfaction did not differ in trials with formal between-group comparison,^11^^,^^13^^,^^15^^,^^16^ although one reported significantly higher self-reported enjoyment with VR on a single-item scale.^10^ Within-group VR-specific satisfaction was uniformly high where reported.^10^^,^^12^^,^^15^^,^^16^ VR-related adverse events were uncommon and self-limited: in the largest awake hand surgery trial, 7 of 59 participants (12%) reported minor symptoms including dizziness, blurred vision, difficulty concentrating and general discomfort;^16^ an earlier trial reported three episodes of dizziness and two of nausea among 21 VR participants, without statements on overlap.^10^ No serious VR-related adverse events occurred in any trial.

## Discussion

This review synthesised the randomised evidence on perioperative VR in adult hand and wrist surgery. The principal findings were a lack of consistent analgesic benefit, a signal of reduced intraoperative anxiety in some awake procedures, and low certainty of evidence for both primary outcomes.

### Pain

Pain outcomes were heterogeneous. In the emergency department, VR was associated with lower pain during laceration repair,^14^ although this trial used simple lidocaine infiltration in an emergency rather than elective population, broadening external validity at the cost of homogeneity. Across five elective trials under regional or local anaesthesia, no significant between-group differences in intraoperative or postoperative pain were observed.^11^, ^12^, ^13^^,^^15^^,^^16^

Hoxhallari et al.^10^ reported lower pain during local anaesthetic injection among participants with high baseline anxiety in a post hoc, graphically presented subgroup analysis, in a trial at high risk of bias; this should be regarded as exploratory.^10^ Where dense regional or local blockade produces minimal intraoperative pain, scope for further distraction-mediated analgesia may be limited.^17^^,^^18^ This ceiling-effect explanation has limits, however: pain during ultrasound-guided block placement in Nijs et al.^16^ was moderate and not at a ceiling, yet was not reduced by VR (mean difference −0.3, 95% CI −1.2 to 0.5; p = 0.22). VR may therefore have limited analgesic effect on brief noxious procedural stimuli, irrespective of subsequent surgical analgesia.

In broader surgical populations, VR's analgesic effect is modest and inconsistent: pooled estimates are low-certainty and below the minimal clinically important difference,^6^ and several trials show no analgesic benefit despite anxiolysis.^19^^,^^20^ This fits meta-regression evidence that VR relieves mainly moderate-to-severe pain,^21^ unlikely once a dense block is established. In awake elective hand and wrist surgery, anxiety rather than pain therefore appears the more tractable target for VR.

### Anxiety

Three of the six trials reported significant reductions in intraoperative anxiety with VR.^10^^,^^15^^,^^16^ Nijs et al.^16^ found reduced anxiety during surgery but not during block placement, suggesting greatest effect during sustained intraoperative exposure rather than brief stimuli. Matsuoka et al.^15^ reported lower intraoperative and postoperative anxiety during WALANT but no reduction preoperatively, suggesting benefit specific to the active procedural period. Hoxhallari et al.^10^ reported reductions across all time points, but the analysis was exploratory and at high risk of bias. By contrast, McConnell et al.^11^ found no significant difference on the APAIS despite a modest reduction in intraoperative diastolic blood pressure, and Xiao et al.^12^ reported numerically higher anxiety with VR that did not reach significance, further complicating the directional signal. Instrument choice may contribute, but population and procedural differences are likely more substantive. Anxiety measurement is also confounded where sedation was used. Faruki et al.^13^ assessed intraoperative anxiety only by postoperative recall, yet control participants reported essentially no recollection of theatre (median agreement 0% vs 97% in the VR group), while Xiao et al.^12^ measured anxiety in real time but note that midazolam given to controls may itself have lowered their scores; these two trials should therefore be read separately from the fully awake pathways.

The anxiolytic effect of VR operates mainly through attentional distraction and cognitive immersion: by occupying the limited attentional resources of conscious patients, VR reduces the bandwidth available to process anxiety-provoking stimuli.^22^, ^23^, ^24^ This depends on the patient remaining cognitively engaged and is therefore attenuated where sedation ablates the cognitive substrate required for distraction-mediated anxiolysis. Wide-awake local anaesthesia is a setting in which this mechanism can operate without pharmacological interference, and the positive intraoperative findings of Matsuoka et al.^15^ and Nijs et al.^16^ in this context support the framework.

### Secondary outcomes and feasibility

Two trials suggested a sedative-sparing effect where intravenous sedation was used,^12^^,^^13^ consistent with meta-analytic evidence that VR may reduce intraprocedural sedation, though effects on opioids remain unclear.^8^ This signal is relevant to ambulatory pathways that include sedation but has limited bearing on pure WALANT, where sedation is intentionally avoided. Physiological responses were heterogeneous, with isolated between-group differences in heart rate and blood pressure rather than consistent changes,^11^^,^^14^^,^^15^ suggesting context-dependent effects (Supplement S1). VR was well tolerated, with no serious adverse events and only infrequent, self-limiting minor effects (dizziness, transient nausea, oculomotor discomfort) in a minority of participants.^10^^,^^16^

### VR immersion and surgeon–patient communication during WALANT

A consideration unique to hand and wrist surgery is the tension between VR immersion and the surgeon–patient interaction on which wide-awake techniques rely. WALANT depends on intraoperative communication: patients move fingers on command for tendon checks, report inadequate anaesthesia and engage with the team.^25^ A patient in a head-mounted display with noise-cancelling headphones is less available for this, and in one trial some participants would have preferred to talk with the surgeon rather than wear the headset.^10^ A meaningful minority may decline immersion: 5 of 52 patients allocated to VR chose not to use it intraoperatively.^11^ Reassuringly, cooperation with instructions to move the hand was easy and immediate among VR users,^10^ though the device adds demands on staff, who must fit, adjust and troubleshoot it while operating.^15^ Protocols incorporating VR during WALANT may therefore need to allow brief headset removal at tendon checks, flexion–extension testing or communication points, particularly in flexor tendon repairs, tenolyses and nerve procedures where active cooperation is central to the awake approach.

Beyond communication, VR adds practical demands on the operative pathway: preoperative orientation and headset fitting, intraoperative adjustment and programme selection, troubleshooting of device faults, and inter-patient cleaning under infection-control supervision.^14^^,^^15^ In the largest awake trial, set-up required little additional time, although adverse effects occasionally necessitated early headset removal and brief clinical intervention;^16^ in another, dedicated research staff managed the equipment, so the demand on routine surgical staff was not captured.^15^ Equipment and software costs, staff training and maintenance are further considerations.^15^ In high-throughput outpatient and procedure-room settings, where short operative times underpin the efficiency case for wide-awake surgery, these demands may materially affect deliverability, and cost-effectiveness was not formally evaluated in any included trial.

### Risk of bias and certainty of evidence

As detailed above, one trial was at low risk of bias,^16^ most had some concerns, and two were at high risk,^10^^,^^14^ chiefly from missing outcome data and, for Hoxhallari et al., deviations from the intended intervention. Under GRADE, certainty was low for both primary outcomes: for pain, downgraded for risk of bias and indirectness from heterogeneous outcome constructs, time points and settings; for anxiety, downgraded for inconsistency and indirectness from variable instruments and timing, despite a directional signal of reduced intraoperative anxiety in some awake contexts.

### Clinical implications

For hand surgeons performing awake procedures, VR may serve as a selective, optional adjunct for intraoperative anxiety during WALANT or regional anaesthesia. The signal is strongest for intraoperative anxiety rather than pain, is heterogeneous, and is not explained by instrument type alone — the single trial using the multi-item APAIS found no difference. Although several trials demonstrated reductions in intraoperative anxiety, only three of the six trials assessing anxiety found a statistically significant benefit, the overall certainty of evidence remains low, and routine clinical implementation cannot yet be recommended. Current evidence therefore supports neither routine adoption nor analgesic benefit beyond effective blockade.

### Limitations and future directions

This review has limitations. Included trials were heterogeneous in design, intervention and outcome assessment, with VR ranging from consumer head-mounted displays to a medical-grade hypnotic system — a spectrum of immersion that may itself influence effect. Although modest in number, the included trials represent the totality of randomised evidence specific to adult hand and wrist surgery, providing procedure-specific evidence previously embedded within broader cross-specialty reviews^6^^,^^26^. The search covered MEDLINE, Embase and CENTRAL; nursing- and psychology-oriented databases were not searched, and although Google Scholar and reference lists were screened, trials indexed only elsewhere may have been missed. Small samples and heterogeneous anxiety instruments precluded meta-analysis. Combining sedation and fully awake trials also adds heterogeneity to the anxiety synthesis, since sedation both attenuates the mechanism by which VR is thought to act and complicates its measurement — a principal reason for our recommendation that future trials restrict enrolment to a single anaesthetic modality. Although eligibility was intentionally broad across procedure type and anaesthetic modality, the identified trials predominantly enrolled patients undergoing minor soft-tissue procedures. Bony procedures such as fracture fixation, arthroplasty or arthrodesis appeared only as small subgroups within mixed cohorts and were never analysed separately; no trial reported outcomes stratified by procedure type, evaluated distal radius fixation, or included wrist arthroscopy or major hand trauma. The findings may therefore not generalise to these settings. Some trials had baseline imbalances (e.g. a younger VR group in Xiao et al.^12^), and the largest trial^14^ did not assess anxiety, broadening external validity but adding population heterogeneity. Reporting of psychiatric background was also inconsistent: three trials reported baseline proportions with diagnosed or treated anxiety or depression,^10^^,^^12^^,^^13^ two reported no psychiatric data,^11^^,^^15^ and three applied psychiatric exclusion criteria of varying stringency.^12^^,^^14^^,^^16^ Diagnosed psychiatric history could therefore not be examined systematically as an effect modifier across trials. Publication bias and selective reporting could not be formally assessed given the few trials per outcome.

Future trials should be designed specifically to reduce the heterogeneity that currently prevents pooling, addressing population, intervention, comparator and outcome in turn. Populations should be defined narrowly and reported in full: trials should specify and, where feasible, restrict enrolment to a single anaesthetic modality (wide-awake local anaesthesia, ultrasound-guided regional anaesthesia, or local anaesthesia with sedation), because the anxiolytic mechanism of VR depends on preserved cognition and is attenuated by sedation. As the anxiety signal is concentrated in awake procedures, wide-awake and regional pathways are the most promising settings. Because baseline anxiety appears to modify the effect, it should be measured at screening and used either to stratify randomisation or to enrich the sample with clinically anxious patients, in whom benefit is most plausible.^10^ The intervention and comparator require standardisation. Trials should report the device, degree of immersion and content, and, critically, the timing and duration of VR relative to the key anxiety-provoking steps (local anaesthetic injection, block placement and intraoperative manipulation), since benefit appears tied to sustained active exposure rather than to brief stimuli. To distinguish the specific benefit of immersive VR from that of simpler, non-immersive distraction, comparators should extend beyond usual care to an active control such as noise-cancelling headphones with music, which has itself reduced intraoperative anxiety during wide-awake hand surgery.^2^ Outcomes should be prespecified and harmonised: a single standardised, multi-item anxiety instrument (such as the APAIS or State-Trait Anxiety Inventory) should serve as the primary endpoint, assessed at defined perioperative time points anchored to active VR exposure and with the intraoperative period as the primary time point, avoiding single-item scales and graphical-only reporting. Trials should be powered for anxiety as the primary outcome rather than treating it as secondary, and should report cybersickness and tolerability using a standardised measure. Adoption of a core outcome set for perioperative VR would further enable future synthesis.

Designs specific to hand and wrist surgery should predefine headset-removal points for tendon checks, flexion–extension testing and nerve assessment, and should capture the implementation outcomes that determine deliverability: staff burden, procedure time, infection-control and device-cleaning workflows, cost-effectiveness, and patient preference for surgeon contact versus immersion.

## Conclusion

Perioperative VR may reduce intraoperative anxiety in awake hand and wrist surgery, but findings are heterogeneous and do not support routine implementation. Analgesic benefit beyond effective local or regional blockade was not demonstrated. VR is best considered an optional patient-preference adjunct, with attention to the trade-off with the intraoperative surgeon–patient communication central to wide-awake techniques. Larger, adequately powered trials using standardised validated anxiety instruments are needed before routine adoption.

## Author contributions

Shivam Bhindi: Conceptualization, Methodology, Investigation, Data curation, Formal analysis, Writing – original draft. Thanmay Mangala Sachidananda: Conceptualization, Methodology, Investigation, Data curation, Formal analysis, Writing – original draft. Ali Alseneid: Investigation, Writing – review & editing. Gunjeet Dua: Conceptualization, Supervision, Writing – review & editing. All authors approved the final version of the manuscript and agree to be accountable for all aspects of the work.

## Ethics

Ethical approval was not required for this systematic review of previously published studies, which did not involve human participants. Informed consent and consent for publication were therefore not applicable, and the review does not contain any individual person’s data, images, or videos.

## Declaration of generative AI and AI-assisted technologies in the manuscript preparation process

During the preparation of this work the authors used Claude (Anthropic) to a limited extent to improve the language and readability of selected portions of the manuscript. After using this tool, the authors reviewed and edited the content as needed and take full responsibility for the content of the published article.

## Funding

This research did not receive any specific grant from funding agencies in the public, commercial, or not-for-profit sectors.

## Guarantor

Shivam Bhindi is the guarantor for this work.

## Data availability

All data analysed in this systematic review are contained within the included published studies, the tables and the Supplementary Material. The structured data-extraction form is available from the corresponding author on reasonable request.

## Declaration of competing interest

The authors declare that they have no known competing financial interests or personal relationships that could have appeared to influence the work reported in this paper.

## Appendix Supplementary materials

S1. Supplementary tables. Secondary outcomes across included randomised controlled trials (Table S1).

S2. Supplementary tables. Patient experience, satisfaction, immersion and tolerability outcomes across included randomised controlled trials (Table S2).

S3. PRISMA 2020 checklist.

S4. Full database search strategies.

S5. Summary of findings table.

## Footnotes

Supplementary material associated with this article can be found, in the online version, at doi:10.1016/j.jpra.2026.09.005.
