# Supplementary material for: Perioperative virtual reality for pain and anxiety in adult hand and wrist surgery: a systematic review
Source: JPRAS Open. 2026 Sep 12;52:532–42. doi: 10.1016/j.jpra.2026.09.005 (PMC13639975; doi:10.1016/j.jpra.2026.09.005)
Supplement: Supplementary file 2 [file mmc2.docx]

**S4. Full Database Search Strategies**

**Medline Search Strategy**

Database: **Ovid MEDLINE® ALL (1946–present)**
Date searched: 19 December 2025
Results retrieved: 64

1. Virtual Reality/
2. (virtual reality or VR or immersive virtual reality or virtual environment* or head-mounted display*).ti,ab.
3. 1 OR 2
4. (hand or wrist).ti,ab.
5. (surger* or procedure* or operation*).ti,ab.
6. 4 AND 5
7. 3 AND 6
8. randomized controlled trial.pt.
9. controlled clinical trial.pt.
10. randomized.ab.
11. randomised.ab.
12. trial*.ab.
13. 8 OR 9 OR 10 OR 11 OR 12
14. 7 AND 13
15. exp animals/ not humans.sh.
16. 14 NOT 15

**Embase (Ovid) Search Strategy**

**Database:** Embase (Ovid) **Coverage:** 1974 to present
Date searched: 19 December 2025
Results retrieved: 200

1. virtual reality/
2. (virtual reality or VR or immersive virtual reality or virtual environment* or head-mounted display*).ti,ab.
3. 1 OR 2
4. hand surgery/
5. wrist surgery/
6. 4 OR 5
7. (hand or wrist).ti,ab.
8. (surger* or surgical or operative or operation*).ti,ab.
9. 7 AND 8
10. 6 OR 9
11. 3 AND 10
12. randomized controlled trial/
13. controlled clinical trial/
14. random*.ti,ab.
15. trial*.ti,ab.
16. 12 OR 13 OR 14 OR 15
17. 11 AND 16
18. animal/ not human/
19. 17 NOT 18

**CENTRAL Search Strategy**

Database: CENTRAL
Date searched: 19 December 2025
Results retrieved: 210

("virtual reality" OR VR OR "immersive virtual reality" OR "virtual environment*" OR "head mounted display*" OR HMD*) AND ("hand surgery" OR "wrist surgery" OR hand OR wrist) AND (surgery OR surgical OR operative OR operation*)
