# Supplementary material for: Perioperative virtual reality for pain and anxiety in adult hand and wrist surgery: a systematic review
Source: JPRAS Open. 2026 Sep 12;52:532–42. doi: 10.1016/j.jpra.2026.09.005 (PMC13639975; doi:10.1016/j.jpra.2026.09.005)
Supplement: Supplementary file 3 [file mmc3.docx]

S5. Summary of Findings Table

| **Perioperative Virtual Reality for Pain and Anxiety During Awake Hand Surgery Under Regional or Local Anaesthesia** | | | |  |
| --- | --- | --- | --- | --- |
| **Patient or population:** reduction of perioperative pain/ anxiety  **Setting:** Adult patients undergoing hand or wrist surgery  **Intervention:** Perioperative virtual reality (VR) intervention  **Comparison:** Standard perioperative care | | | |  |
| Outcomes | Impact | № of participants (studies) | Certainty of the evidence (GRADE) |  |
|  |  |  |  |  |
| Pain intensity (primary study timepoint; mixed acute peri-procedural and longer-term constructs) (Pain) assessed with: Numeric Rating Scale (NRS), Visual Analogue Scale (VAS), Wong–Baker FACES scale, Likert-type scales, observational pain tools (CPOT), and PROMIS Pain Interference (change score), assessed at the primary perioperative timepoint reported by each study. | Across elective perioperative settings, most trials reported no clear between-group difference in pain at their primary perioperative timepoint. One emergency department trial reported lower immediate post-procedure pain with VR (patient-reported VAS and CPOT). One trial reported subgroup-only benefit for injection pain (higher baseline anxiety subgroup), with pain values otherwise reported graphically. | (7 RCTs) | ⨁⨁◯◯ Low^a,b^ |  |
| Anxiety (perioperative/intraoperative; heterogeneous measures and timing) (Anxiety) assessed with: NRS anxiety (0–10), NVAAS (0–10), VAS anxiety (0–100), APAIS (change score), and a non-validated 0–100 agreement-style item reflecting intraoperative anxiety. | Across perioperative procedural settings, several trials reported lower anxiety with VR during awake procedures, particularly during intraoperative assessment. Other trials reported no clear between-group difference. Overall interpretation is limited by heterogeneity in anxiety measurement, including the use of different validated scales, change scores, and a non-validated agreement-style item, as well as variation in assessment timing (block placement, intraoperative, and immediate post-procedure or retrospective assessment). | (6 RCTs) | ⨁⨁◯◯ Low^c,d^ |  |
| ***The risk in the intervention group** (and its 95% confidence interval) is based on the assumed risk in the comparison group and the **relative effect** of the intervention (and its 95% CI).  **CI:** confidence interval | | | |  |
| **GRADE Working Group grades of evidence** **High certainty:** we are very confident that the true effect lies close to that of the estimate of the effect. **Moderate certainty:** we are moderately confident in the effect estimate: the true effect is likely to be close to the estimate of the effect, but there is a possibility that it is substantially different. **Low certainty:** our confidence in the effect estimate is limited: the true effect may be substantially different from the estimate of the effect. **Very low certainty:** we have very little confidence in the effect estimate: the true effect is likely to be substantially different from the estimate of effect. | | | |  |

#### Explanations

a. Downgraded one level for risk of bias: two trials contributing pain data were judged high overall risk of bias (Gharanli 2024; Hoxhallari 2019) and most remaining trials had some concerns, with only one trial judged low risk overall (Nijs 2025).

b. Downgraded one level for indirectness: pain was measured using non-comparable instruments and clinically distinct time horizons/settings (acute procedural pain measures, observational CPOT, and 2-week PROMIS Pain Interference; ED vs elective perioperative settings), limiting applicability to a single clearly defined pain construct.

c. Downgraded one level for inconsistency: results varied across trials, with reductions in anxiety reported in some intraoperative assessments but no clear differences in others; effects were not consistent across measures or study contexts.

d. Downgraded one level for indirectness: anxiety was assessed using heterogeneous instruments (including a non-validated agreement-style item and change scores) and differing assessment timings (block placement, intraoperative, and immediate post-procedure/retrospective assessment), limiting confidence that all studies address a single comparable anxiety outcome.
