# Supplementary material for: Perioperative virtual reality for pain and anxiety in adult hand and wrist surgery: a systematic review
Source: JPRAS Open. 2026 Sep 12;52:532–42. doi: 10.1016/j.jpra.2026.09.005 (PMC13639975; doi:10.1016/j.jpra.2026.09.005)
Supplement: Supplementary file 4 [file mmc4.docx]

**Table S1. Secondary Outcomes Across Included Randomised Controlled Trials**

| **Study (Author, Year)** | **Domain** | **Outcome (specific)^a,b^** | **Instrument or unit** | **Primary timepoint reported** | **N analysed (VR/Control)** | **VR group result** | **Control group result** | **Statistical comparison** | **Notes** |
| --- | --- | --- | --- | --- | --- | --- | --- | --- | --- |
| **Faruki et al., 2022** | Sedation requirements | Propofol requirement | Propofol dose rate (mg·hr⁻¹), mean (SD) (95% CI) | Intraoperative | 17 / 17 | 125.3 (SD 296.0) (95% CI 10.3–267.9) | 750.6 (SD 334.6) (95% CI 598.6–911.5) | p < 0.001 | Intraoperative dosing was the prespecified primary focus of this RCT |
| **Xiao et al., 2025** | Analgesic requirements | Fentanyl dose | mcg, mean (min–max) | Intraoperative | 20 / 21 | 10 (0–100) | 27.4 (0–200) | p = 0.02 | Statistically significant reduction in opioid requirement. |
| **Xiao et al., 2025** | Sedation requirements | Midazolam dose | mg, mean (min–max) | Intraoperative | 20 / 21 | 0.4 (0–4) | 1.2 (0–4) | p = 0.04 | Statistically significant reduction in benzodiazepine requirement |
| **Xiao et al., 2025** | Sedation requirements | Propofol dose | mg, mean (min–max) | Intraoperative | 20 / 21 | 26.3 (0–401.2) | 44.5 (0–323.9) | p = 0.54 | Propofol use was discretionary and not protocolised; no between–group difference observed. |
| **Faruki et al., 2022** | Recovery efficiency | PACU length of stay | minutes, median (Q1–Q3) | PACU | 17 / 17 | 53.0 (43.0–72.0) | 75.0 (57.5–89.0) | p = 0.018 | Shorter PACU LOS in VR group. |
| **Xiao et al., 2025** | Recovery efficiency | PACU recovery time | minutes, mean (min–max) | PACU | 20 / 21 | 39.4 (17–70) | 46.3 (15–140) | p = 0.39 | No between–group difference |
| **Faruki et al., 2022** | Functional outcome | Upper–limb disability | Postoperative QuickDASH score, median (Q1–Q3) | Postoperative | 17 / 17 | 20.5 (9.1–40.9) | 40.9 (27.3–59.1) | p = 0.056 | Trend toward lower disability; not statistically significant. |
| **Gharanli et al., 2024** | Physiologic measures | Systolic blood pressure | mmHg, mean (SD) | Immediately postprocedure | 80 / 80 | 123.15 (SD 8.86) | 126.60 (SD 9.83) | p = 0.02 | Baseline (preprocedure) SBP did not differ. Other vitals largely NS. |
| **Gharanli et al., 2024** | Physiologic measures | Respiratory rate | breaths per minute, mean (SD) | Immediately postprocedure | 80 / 80 | 16.84 (SD 0.96) | 17.40 (SD 0.85) | p < 0.001 | Baseline respiratory rate did not differ. |
| **Matsuoka et al., 2025** | Physiologic measures | Heart rate | bpm, mean (SD) | Intraoperative | 30 / 29 | 72 (SD 10.1) | 83 (SD 16.69) | p = 0.04 | BP was NS at all timepoints in this trial. |
| **Matsuoka et al., 2025** | Physiologic measures | Heart rate | bpm, mean (SD) | Postoperative | 30 / 29 | 73 (SD 9.53) | 84 (SD 15.57) | p = 0.03 | Postop HR remained lower in VR group. |
| **McConnell et al., 2025** | Physiologic measures | Diastolic blood pressure (intraoperative minus preoperative) | mmHg, median (Q1-Q3) | Intraoperative | 45 / 45 | **NR** (reported graphically) | **NR** (reported graphically) | p = 0.003 | Diastolic blood pressure reported as change from preoperative baseline. Numerical values were presented graphically and could not be extracted. |

^a^Physiologic and clinically actionable secondary outcomes are selectively tabulated to highlight outcomes with potential relevance to perioperative management. All reported secondary outcomes, including those without statistically significant between-group differences, are summarised descriptively in the Results section for completeness.

^b^Outcomes demonstrating within–group change without statistically significant between–group differences were not tabulated.

Abbreviations: HR, heart rate; IQR, interquartile range; RA, regional anaesthesia; RR, respiratory rate; SD, standard deviation; SpO₂, peripheral oxygen saturation; VR, virtual reality; PACU, postanaesthesia care unit.
