# Supplementary material for: Perioperative virtual reality for pain and anxiety in adult hand and wrist surgery: a systematic review
Source: JPRAS Open. 2026 Sep 12;52:532–42. doi: 10.1016/j.jpra.2026.09.005 (PMC13639975; doi:10.1016/j.jpra.2026.09.005)
Supplement: Supplementary file 5 [file mmc5.docx]

**Table S2. Patient Experience, Satisfaction, Immersion, and Tolerability Outcomes Across Included Randomised Controlled Trials**

| **Study (Author, Year)** | **Outcome domain** | **Outcome (specific)^a^** | **Instrument / scale** | **Assessment timepoint** | **N analysed (VR/Control)** | **VR group result^b^** | **Control group result** | **Statistical comparison^c^** | **Notes** |
| --- | --- | --- | --- | --- | --- | --- | --- | --- | --- |
| **Faruki et al., 2022** | Patient experience | “I remember being aware of how I felt in the OR” | 0–100 scale, median (Q1–Q3) | Perioperative | 17 / 17 | 97 (79–100) | 0 (0–0) | p < 0.001 | Higher scores reflect greater recall of intraoperative awareness, as reported. |
| **Faruki et al., 2022** | Patient experience | “I felt relaxed” | 0–100 scale, median (Q1–Q3) | Perioperative | 17 / 17 | 99 (79–100) | 100 (100–100) | p = 0.06 | No statistically significant between-group difference. |
| **Faruki et al., 2022** | Patient experience | “Interested in VR for future surgery” | 0–100 scale, median (Q1–Q3) | Perioperative | 17 / 17 | 100 (69–100) | 100 (68–100) | p = 0.68 | Ceiling effect likely. |
| **Faruki et al., 2022** | Patient satisfaction | Overall satisfaction with procedure | 0–100 scale, median (Q1–Q3) | Perioperative | 17 / 17 | 92 (77–100) | 100 (100–100) | p = 0.09 | No statistically significant between-group difference. |
| **Hoxhallari et al., 2019** | Patient experience | Enjoyment of experience | 10-point Likert scale, median | Postprocedure questionnaire | 21 / 20 | 8 (median on 10-point Likert scale) | 3 (median on 10-point Likert scale) | p < 0.01 | Median enjoyment score reported descriptively; full distribution not provided. |
| **Nijs et al., 2025** | Patient satisfaction | Satisfaction during RA placement | NRS 0–10 (median, IQR) | During RA block placement | 59 / 59 | 9.0 (8.0–10.0) | 9.0 (8.0–10.0) | p = 0.44 | No between-group difference |
| **Nijs et al., 2025** | Patient satisfaction | Satisfaction during surgery | NRS 0–10 (median, IQR) | Intraoperative | 59 / 59 | 10.0 (9.0–10.0) | 9.0 (9.0–10.0) | p = 0.70 | No between-group difference |
| **Nijs et al., 2025** | Patient satisfaction | Global satisfaction with VR therapy | NRS 0–10 (median, IQR) | Perioperative | 59 / 59 | 9.0 (8.0–10.0) | Not assessed | Not applicable | Outcome assessed in VR group only by study design; no comparator available. |
| **McConnell et al., 2025** | Patient satisfaction | Overall postoperative surgical satisfaction | SSQ-8 (total score) | Postoperative | 45 / 45 | NR | NR | p = 0.313 | Numeric SSQ-8 totals not reported; authors state no between-group difference. |
| **Matsuoka et al., 2025** | Patient satisfaction | Satisfaction with WALANT | 0–10 scale (mean (SD)) | Perioperative | 30 / 29 | 9.7 (SD 0.83) | 9.8 (SD 0.49) | p = 0.74 | High satisfaction in both groups |
| **Matsuoka et al., 2025** | Patient satisfaction | Satisfaction with VR | Categorical | Perioperative | 30 / 29 | Very satisfied: 21 (70%)  Satisfied: 8 (26.6%)  Dissatisfied: 1 (3.3%)  Very dissatisfied: 0 | Not assessed | Not applicable (VR-only outcome) | Category reporting used |
| **Nijs et al., 2025** | VR immersion | Presence/immersion | IPQ total (0–6), mean (SD) | Intraoperative(during VR exposure) | 59 / 59 | 4.2 (SD 0.9) | Not assessed | Not applicable (VR-only outcome) | IPQ subscales also reported; total chosen for concision. |
| **Nijs et al., 2025** | Tolerability and safety | Adverse VR effects | Event reporting | Event reporting | 59 / 59 | 7 patients reported adverse VR-related effects | Not assessed | Not applicable (VR-only outcome) | Effects included discomfort, focusing difficulty, salivation, sweating, blurred vision, dizziness, vertigo (as reported) |
| **Hoxhallari et al., 2019** | Tolerability and safety | Cybersickness symptoms | Event reporting | During/after VR | 21 / 20 | Dizziness 3; nausea 2; vomiting 0 | Not assessed | Not applicable (VR-only outcome) | VR delivery failed on five occasions due to device overheating (Samsung Galaxy S7), leading to exclusion of five participants from analysis. |
| **Xiao et al., 2025** | Patient experience | Feeling relaxed during surgery | Post-treatment survey | Intraoperative(postoperative recall) | 20 / 21 | 75% (15/20) agreed | Not assessed | Not applicable (VR-only outcome) | VR-only item |
| **Xiao et al., 2025** | VR immersion | The VR experience effectiveness for distracting from surroundings | Post-treatment survey | Perioperative(postoperative recall) | 20 / 21 | 65% (13/20) agreed | Not assessed | Not applicable (VR-only outcome) | VR-only item |

^a^Patient experience, satisfaction, immersion, and tolerability outcomes were reported heterogeneously across studies and frequently described descriptively.

^b^Outcomes assessed only in the VR group reflect study design without a comparator.

^c^Absence of statistical comparison indicates non-comparative reporting rather than missing data.

Abbreviations: IPQ, Igroup Presence Questionnaire; IQR, interquartile range; NRS, numeric rating scale; RA, regional anaesthesia; SSQ-8, Surgical Satisfaction Questionnaire-8; VR, virtual reality; WALANT, wide-awake local anaesthesia no tourniquet.
